# Self-mixed antibiotic bone cement: western countries learn from developing countries

**DOI:** 10.3109/17453670903317122

**Published:** 2009-10-01

**Authors:** Geert H I M Walenkamp

**Affiliations:** Caphri Research Institute, Maastricht University Medical Centrethe Netherlandsg.walenkamp@mumc.nlg.h.walenkamp@home.nl

The paper of Rasyid et al. (2009) in this issue of Acta Orthopaedica addresses a problem that is important for those who treat bone and joint infections in developing countries, but increasingly also in so-called western countries. The paper is interesting and helpful for all surgeons who have to make antibiotic-loaded beads and spacers by themselves. This is necessary when commercially produced bone cement and beads are not available, or when the antibiotic in the PMMA is not effective due to resistance of the causative bacteria. The paper is based on a recent thesis from the research group of the Department of Biomedical Engineering at the University of Groningen, and is part of a series of publications about basic and clinical research on antibiotic-loaded cement and other biomaterials (Belt van de 2001, Hendriks 2003, Neut 2003, Ensing 2006, Engelsman 2009, Rasyid 2009).

When gentamicin-loaded bone cement (Refobacin-Palacos) became available in Germany in the 1970s, efforts to treat osteomyelitis by filling the debrided cavity with solid plugs of antibiotic-loaded bone cement were not successful in patients in France and Germany, nor in animal experiments in the Mayo Clinics (Vidal and Allieu 1969, Voorhoeve and Stöhr 1973, Jenny et al. 1977, Fitzgerald 1983). Thus, Klaus Klemm started to make hand-made beads in his infection unit in the BG Unfallklinik in Frankfurt (Klemm 1993). The argument at that time for developing beads instead of solid cement plugs was that the debrided osteomyelitic cavities had to secrete freely—and it was not the increase in surface area-volume ratio, as is the accepted explanation for improved effectiveness nowadays. The Kulzer company (Wehrheim, Germany), the producer of Refobacin-Palacos, developed the production of gentamicin-PMMA beads (Septopal) in the seventies. Septopal beads have now been on the market for more then 30 years, a remarkably long time for a pharmacological product. This shows that the gentamicin-PMMA beads are still a unique product; they are needed in clinical practice and there is no good alternative when there is a requirement for local antibiotic treatment. Over the first 25 years, Septopal was distributed in most countries of Europe by Merck (Darmstadt, Germany), and nowadays it is distributed by Biomet (Biomet Europe, Berlin, Germany). It is still not accepted by the FDA in the US.

One of the arguments against the use of antibiotic-loaded beads is the lack of proof of effectivity in good randomized clinical trials (RCTs). The most cited RCT was performed in the US in an attempt to get FDA approval, in 380 patients with osteomyelitis: systemic antibiotic treatment versus gentamicin beads. However, the protocol was violated because surgeons worried about withholding systemic antibiotics from patients who were treated with beads. In this trial, however, the costs were lower when beads were used, and there were less systemic adverse events with antibiotics when local antibiotics were used (as compared to treatment with systemic antibiotics) (Blaha et al. 1993). Another RCT in the US studied the treatment of infected total hip and knee prostheses in 28 patients, comparing 6 weeks of local treatment with gentamicin-PMMA beads with systemic antibiotic therapy. No statistically significant difference in infection healing was found, but that could have been due to the fact that the reimplantations in both groups were performed without the use of antibiotic in the bone cement (Nelson et al. 1993).

An RCT was even performed in a developing country. This rarely cited study was done in an isolated Himalayan region of Nepal. Lars Lindberg (at that time from Kristianstad, Sweden) and Margareta Höök treated 45 patients with debridement followed by the use of gentamicin beads or open treatment package of the debrided cavity (Höök and Lindberg 1987). The randomization was, however, imperfect due to the experience that “the immediate good results with gentamicin beads were so conspicuous that it was impossible to withhold this treatment from the children and the severe cases”, as they stated. Finally, I started an RCT in 1977, comparing gentamicin-PMMA beads with suction drainage and systemic antibiotics in osteomyelitis and infected total hip. The trial was stopped after 27 patients because of the advantages of the bead therapy regarding nurses' workload, primary wound healing, and avoidance of superinfection compared to suction drainage (Walenkamp 1983).

In the developing countries surgeons also became aware of the benefit of local antibiotic treatment of bone infections. The Septopal beads were, however, too expensive and surgeons had to look for alternative solutions. Beads made of plaster of Paris were an important alternative in the beginning.

The use of plaster of Paris in bone defects had been known since 1892, when Trendelenburg in Bonn (Germany) filled bone defects (Peltier 1961). Admixture of gentamicin in plaster of Paris pellets appeared to be pharmacokinetically effective in the treatment of bone infections (Mackey et al. 1982), and antibiotic-loaded plaster of Paris pellets were used in several clinics, e.g. in France (Berck Plage, Paris), Algeria, and India.

For many years, a group of Dutch orthopedic surgeons used preformed gentamicin-containing plaster of Paris beads during their visits to Indonesia and Africa; these were made by a pharmacist at the hospital in Arnhem (Sorge et al. 1989). So gentamicin-loaded plaster of Paris was one of the first applications of resorbable antibiotic carriers, but the results were not excellent and its use was not widespread in Europe. A commercial variant with tobramycin has been available for a few years (Chang et al. 2007).

The production by orthopedic surgeons in Indonesia of hand-made beads was originally done by mixing bone cement with fosfomycin, an antibiotic often used in developing countries (Rasyid 2009). They formed beads of 1.5–3 cm diameter by hand. These relative large beads have a smaller surface area to volume ratio (1–1.5 cm^-1^) than Septopal beads (8.6 cm ^-1^), so the release is worse. The paper of Rasyid et al. describes how a method can be developed to create hand-made gentamicin-loaded PMMA beads with very good pharmacokinetic properties in combination with substantially reduced costs when compared with the commercially available beads (Septopal): 112 USD instead of 350 USD.

These authors used an elegant method to increase the antibiotic release: simply the reduction of the monomer content by 50%, which causes large pores in the incompletely polymerized polymer particles. Porosity is one of the most important factors in the process of sustained release of bone cement (Belt van de et al. 2000). In Septopal beads, the porosity is increased by the addition of glycine as a filler (which gets very little mention in the product information). Rasyid et al. used another filler by admixing the polymer with polyvinylpyrrolidone (PVP), the second reason for improvement of the porosity and therefore of the antibiotic release.

In western countries, improvement of antibiotic release from bone cement is also important. Spacers have a low surface area to volume ratio, resulting in a limited and often insufficient release of antibiotic (Greene et al. 1998, Walenkamp 2007, Moojen et al. 2008). In the literature, spacers are mostly described as being effective in the treatment of infected prostheses and osteomyelitis defects, but there appears to be an important publication bias: all results are good. The release of antibiotics from spacers is much less than from beads, resulting in suboptimal local antibiotic concentrations. This could largely be improved by changing the bone cement, as described in the thesis of Rasyid (2009) and the article of Rasyid et al. (2009).

Antibiotic-loaded beads are hand-made in the US because Septopal is not accepted by the FDA. Most surgeons in the US use tobramycin with Simplex bone cement in hand-making beads (Patzakis et al. 1993, Ostermann et al. 1995). In Europe, the need for hand-made beads has been on the increase for 10 years due to more gentamicin resistance. Vancomycin is generally used to treat resistant strains of *Staphylococcus aureus* and *S. epidermidis*, possibly in combination with gentamicin (Taggart et al. 2002, Kelm et al. 2004, Stockley et al. 2008). Surgeons form the beads by hand and they are mostly held on a wire to form chains, facilitating removal. Self-made beads are generally not as good as commercial beads, however (Neut et al. 2003).

Self-made beads could be avoided by the “patient-matched service” of Biomet (Berlin Germany) that made it possible to order vancomycin-gentamicin PMMA beads for an individual patient, based on the resistance pattern of a causative agent (Pfefferle and Nies 2004). For 5 years, I treated a number of patients with these beads and the measured amount of release of vancomycin in the exudate appeared to be excellent. However, this service stopped suddenly without any warning in October 2008, and now we are also forced to make beads by hand.

Finally, molds are helpful in the production of hand-made beads. Rasyid (2009) describes a template for a mold produced in Indonesia, and made of PTFE in stainless steel. Other molds have been described from the US and are available from the University of Vermont (Goodell et al. 1986, Cunningham et al. 2000, DeCoster and Bozorgnia 2008), as well as from Germany (Kelm et al. 2004).

Surgeons should be aware that beads or spacers that they make themselves must have good pharmacokinetic properties to be effective, and that not all kinds of antibiotics may be released properly from bone cement. With the special technique for improvement of antibiotic release properties of bone cement described in the article of Neut et al., and the use of a mold, it is now possible for the surgeon to make customized antibiotic-loaded PMMA beads with different antibiotics and with an improved antibiotic release. We will need this technique increasingly for prosthetic joint infections and osteomyelitis, since resistant bacteria such as CNS, MRSE and MRSA are becoming ever more frequent. It is remarkable that the scientific help of a western university (Groningen) to solve a problem in a developing country may improve the treatment of bone and joint infections in many centres in the western world itself.
